# Cluster headache suicidality: a systematic review with a meta-analysis

**DOI:** 10.1186/s10194-025-02140-x

**Published:** 2025-12-04

**Authors:** E. K. Van Obberghen, R. Fabre, M Lanteri-Minet

**Affiliations:** 1 Pain Department, FHU InovPain, UR2CA-PIN, CHU Nice and Côte d’Azur University, Nice, France; 2 Public Health Department, UR2CA - RESPECT, CHU Nice and Côte d’Azur University, Nice, France; 3 INSERM U1107 Migraine and Trigeminal Pain, Auvergne University, Clermont-Ferrand, France

**Keywords:** Cluster headache, Suicidal ideation, Suicide attempts, Burden

## Abstract

**Background:**

Suicide is considered as common in patients with cluster headache (CH) and is defined as ‘suicidal headache’. However, the exact level of suicidality is not known and is intuitively assumed to be correlated with the CH severity.

**Methods:**

This work is a systematic review of data accessible through PubMed and published up to July 25, focusing on the suicidality of CH according to the rates of suicidal ideation and suicide attempts. This meta-analysis was carried out with all selected studies then by considering studies with specialized recruitment and studies with non-specialized recruitment. A qualitative analysis was performed to identify determinants of CH suicidality.

**Results:**

Among the 53 publications identified, 12 were selected corresponding to 10 studies. These selected studies included a total of 34180 subjects (range 75-24131). Eight were performed in a specialized field (headache tertiary center, neurology clinic or via CH patients’ association) and 2 in a non-specialized field (via heath registry). The overall rate of suicidal ideation in CH was estimated at 8.0% (95%CI [7.7; 8.3] and overall rate of suicide attempts in CH was estimated at 1.2% (95%CI [1.1; 1.3]). In a non-specialized field, these rates were estimated at 5.2% (95%CI [4.9; 5.4]) and 1.1% (95%CI [1.0; 1.2]) respectively. In specialized field, these rates were estimated at 44.6% (95%CI [42.7; 46.6]) and 5.1% (95%CI [3.9; 6.7]) respectively. The qualitative analysis showed that few determinants have been considered but it appears that the risk is greater during CH attacks, and it involves psychological determinants such as demoralization.

**Conclusion:**

The overall suicidal risk in CH does not appear to be higher than that of the general population, but there is a suicidal risk increase among CH patients followed up in specialized field. This higher risk is indeed probably related to the severity of CH in terms of pain, but it is also probably related to other factors such as impulsive aggressiveness during CH attacks and psychological factors such as demoralization.

**Registration:**

The systematic review protocol was registered with the International Prospective Register of Systematic Reviews (PROSPERO) on 07/22/2025 (number: CRD420251110095).

## Background

Cluster headache (CH), which is the most common of the trigeminal autonomic cephalalgias (TACs), is characterized by attacks of severe to very severe unilateral (orbital, supraorbital and/or temporal) pain lasting from 15 to 180 min (when untreated) associated with ipsilateral autonomic symptoms and/or with restlessness or agitation [1]. Attacks occur once every other day to eight times a day with pain-free periods of at least 3 months in episodic CH (ECH) and without remission or with remissions lasting less than 3 months in chronic CH (CCH) [1]. Due to the severity and recurrence of attacks, this primary headache is referred to as a ‘suicidal headache’ [2] with tragic clinical observations such as that of a 56-year-old male with a history of CCH who, during a nocturnal attack, aimed a shotgun toward his symptomatic eye and fired [3].

It is therefore essential to estimate the rate of suicide attempts associated with the cluster headache. It is also essential to estimate the rate of suicidal ideations in this primary headache because, if most instances of suicidal ideation do not lead to suicide attempts [4], suicidal ideation is a predictor of suicide attempts [5]. Unfortunately, to date, the level of suicidality, considering the rate of suicidal ideation and the rate of suicide attempts, has not been reported in a large representative CH population [6]. It is also likely that suicidality differs according to the severity of the cluster headache, so that interpretation of the results of studies devoted to this component of the CH burden must consider the origin of the patients included in these studies (general population, healthcare claims data, primary care, CH patients’ association websites, neurology, headache tertiary care).

Considering this background, our hypothesis is that the increase in suicidality does not concern all CH patients, but possibly a sub-group of patients for whom it would be important to identify the determinants of this increased suicidality.

To test this hypothesis, we performed a systematic review of data on suicidality associated to CH published up to July 2025. This systematic review includes a meta-analysis considering studies carried out in headache tertiary centers, neurology clinics or via CH patients’ association websites and those with a broader recruitment from the general population, primary care or via healthcare claims data. This systematic review also includes an identification of determinants of suicidality in CH patients.

## Methods

This systematic review was based on the Preferred Reporting Items for Systematic Reviews and Meta-Analyses Protocols (PRISMA-P) guidelines [7]. In accordance with these guidelines, our systematic review protocol was registered with the International Prospective Register of Systematic Reviews (PROSPERO) on 07/22/2025 (registration number: CRD420251110095).

### Search strategy

A comprehensive search on PubMed database was carried out in July 2025. The search terms were ‘suicidality’ OR ‘suicide’ OR ‘suicidal ideation’ OR ‘suicidal tendencies’ OR ‘suicidal thoughts’ OR ‘suicidal plan’ OR ‘suicidal attempt’ OR ‘self-injury’ These were combined with a search for ‘cluster headache’. In addition to this electronic search, we screened the reference lists of the selected articles and relevant literature known by the authors.

Inclusion criteria were: (i) prospective and retrospective studies, case series and survey on suicidality and CH; (ii) adult or children subjects with a diagnosis of cluster headache done by a physician or according to International Classification of Headache Disorders (ICHD) criteria or according to the International Classification of Diseases (ICD); (iii) no restrictions by date; (iv) no restrictions by geographical location; (v) English language articles. Exclusion criteria were: (i) case reports; (ii) adult or children subjects with a CH self-diagnosis; (iii) studies with less than 10 participants. According to these inclusion/exclusion criteria, two authors (EKVO and MLM) independently assessed all title and abstracts for inclusion. Full-text papers were retrieved for articles meeting the eligible criteria and for articles for which these criteria could not be verified solely by the title and abstract. All full-text articles were assessed independently by two authors (EKVO and MLM), and disagreement was resolved by discussion to reach consensus.

### Data extraction

Data were independently extracted by two authors (EKVO and MLM). Data extracted included date, country, study design, recruitment (general population, primary care, healthcare claims data, CH patients’ association website, neurology clinic, headache tertiary center), methods of data acquisition, population (number of participants, age, men: women ratio, percentage of participants with ECH and CCH), method of CH diagnosis, method to suicidality evaluation, rate of suicidal ideations (passive and active), suicidal plan and suicide attempts. The discrepancies were resolved by discussion to reach consensus amongst EKVO and MLM.

### Risk of bias (quality) assessment

Quality assessment of studies selected in this systematic review was performed using the Joanna Briggs Institute (JBI) Appraisal Checklist tool [7] for case series studies and the Oxford Centre for Evidence-Based Medicine (OCEBM) critical appraisal tool [8] for survey studies. The studies were independently assessed by two authors (VEO and MLM) and the discrepancies were resolved by discussion to reach consensus.

### Analysis

This work involved two analyses, one quantitative and one qualitative.

### Quantitative analysis

The quantitative analysis was based on a meta-analysis. For this meta-analysis, we used the rate of suicidal ideation, considering passive suicidal ideation, and the rate of suicide attempts as indicators of suicidality in patients suffering from CH. The rates of suicidal ideation and suicide attempts with the respective 95% confidence interval (CI) were calculated on the combined numbers in the selected studies. Study heterogeneity was performed using I2 (less than 25% viewed as low heterogeneity, between 25% and 50% as moderate, and over 50% as high heterogeneity). We conducted a meta-analysis of all the selected studies, as well as two other meta-analyses, one including studies from specialized recruitment (CH patients’ association website, neurology clinic, headache tertiary center) and the other including studies from non-specialized recruitment (general population, primary care, health registry). The metaprop function in the meta package of R-4.3.0 software was used.

### Qualitative analysis

For the studies in which data were available, the quantitative analysis compared the rates of passive and active suicidal ideation. Similarly, for the studies in which data were available, the quantitative analysis sought to identify possible determinants of suicidality in CH patients.

## Results

### Studies selected

**Fig. 1:**
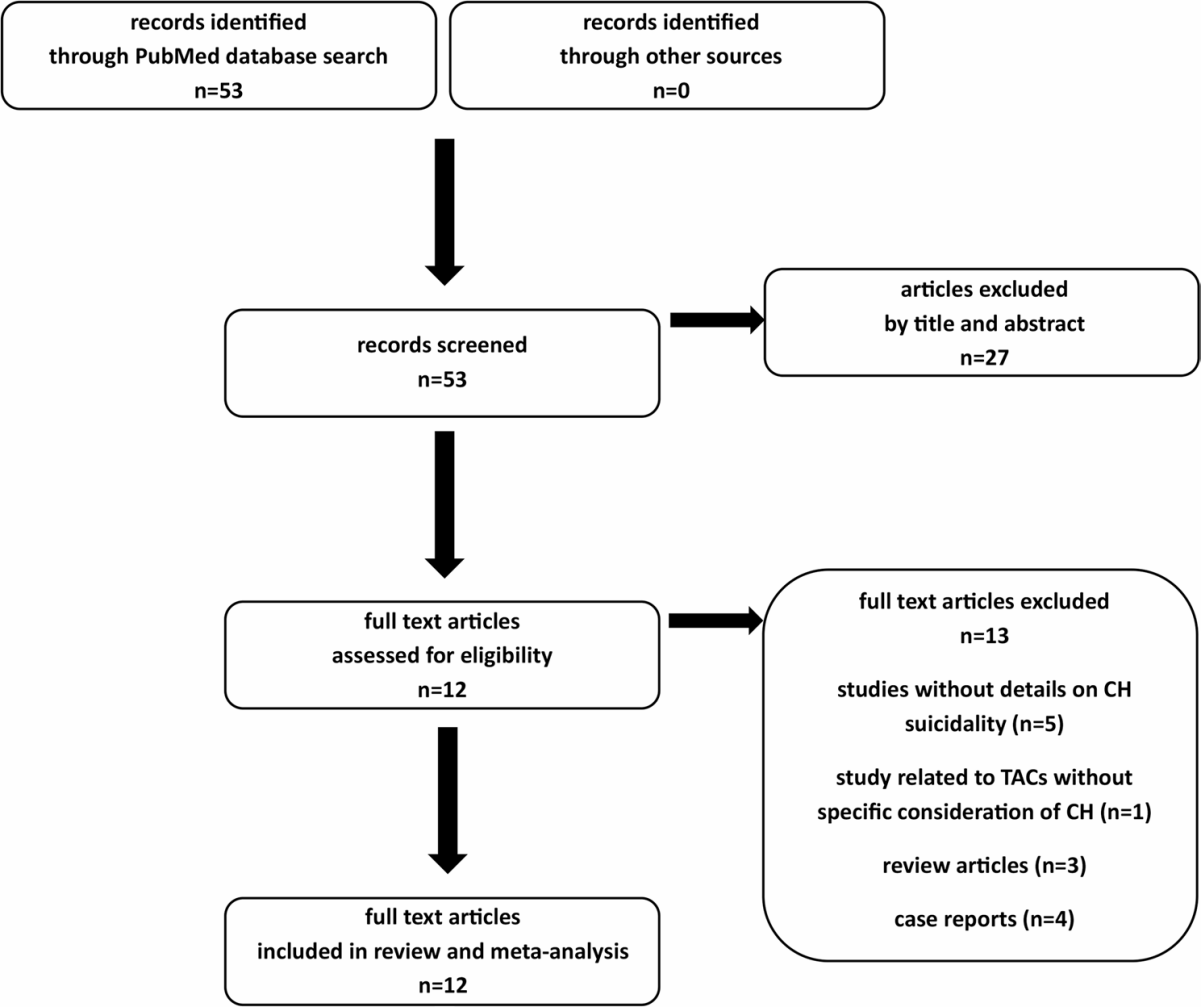
PRISMA flow diagram studies selection (CH: cluster headache/TACs: trigeminal autonomic cephalalgias)

The search carried out on data related to suicidality and CH published up to July 2025 is summarized in the PRISMA flow chart presented in Fig. 1. This search identified 53 unique articles published between February 1952 and April 2025. All articles were screened by title and abstract and 27 articles were excluded at this stage. Full-text articles were assessed for the remaining 25 articles and finally 12 articles, published between April 2011 and April 2025, were selected for the systematic review (Table 1). Among the selected articles, 3 were from the same study [11, 12, 14] and 9 from other studies [10, 13, 15–21]. Studies were survey studies [11–14, 16, 19–21], case series studies [10, 15, 18] and a clinical trial [17]. Two were international studies [17, 20] and the other studies were national studies of which 4 took place in USA [11–14, 16, 19], 2 in Europe [10, 21] and 2 in Asia [15, 18]. Five of the study cohorts were recruited from tertiary headache centers and neurology clinics [10, 15, 17, 18, 21], 3 were recruited through CH patients’ association websites [11, 12, 14, 16, 20] and 2 were recruited through healthcare claims data [13, 19]. These selected studies included a total of 34,180 subjects (range 75-24131). This whole population was composed of 27,703 men, 6195 women and 282 subjects with sex not specified. The form of CH was indicated only 1028 subjects with 586, 331 and 111 corresponding to ECH, CCH and undetermined respectively.

**Table 1:** Description of selected studies (* FF : face-face, I: internet, MR: medical records/**CHPAWS: cluster headache patients’ association website, HR: health registry, N: neurology clinic, THC: tertiary headache center/CCH: chronic cluster headache, ECH: episodic cluster headache/NR: not reported/SPAC: Spanish speaking America countries)

Ref	Authors	Year	Country	Study design	Data acquisition*	Origin of recruitment**	Number of subjects	Age	Men/Women	ECH/CCH/ undetermined	CH diagnosis
[10]	Jürgens et al.	2011	Germany	Case series	FF	THC and NC	75	NR	NR	48/27/0 26 ECH in-bout	ICHD-2
[11] [12] [14]	Rozen and Fischman	2012 2018	USA	Survey	I	CHPAWS	1134	NR	816/318	NR	neurologist
[13]	Choong et al.	2017	USA	Survey	MR	HR	7589	49.97 ± 13.4	4356/3233	NR	ICD-9
[15]	Ji Lee et al.	2019	Corea	Case series	FF	THC	175	38.4 ± 11.1	149/26	154/8/13 all ECH in-bout	ICHD-3
[16]	Koo BB et al.	2021	USA	Survey	I	CHPAWS	100	45.5 ± 12.3	53/47	56/44	ICHD-3
[17]	Lainez et al.	2022	International	Clinical trial	FF	THC	233	44.9 ± 10.9	365/80	0/445/0	ICHD-3
[18]	Kim et al.	2022	Corea	Case series	FF	THC	445	NR	365/80	328/19/98	ICHD-3
[19]	Seng et al.	2024	USA	Survey	MR	HR	24,131	NR	21,763/2368	NR	ICD-9/10
[20]	Calandre et al.	2024	Spain SPAC	Survey	I	CHPAWS	91	44.8 ± 10.8	32/59	NR	physician
[21]	Göbel et al.	2024	Germany	Survey	FF	THC	207	48.16 ± 13.6	n? 62.3%/n? 37.7%	n 38.2%/n? 61.2% all ECH in-bout	ICHD3

### Data extracted

Data extracted in the 12 selected articles related to the 10 considered studies are presented in Table 2. All the studies were considered for the quantitative analysis and among them all reported the rate of suicidal ideations [10–21] whereas only 7 studies reported the rate of suicide attempts [11–19]. The rates of passive and active suicidal ideation were specified in two studies [15, 16] and were considered for the qualitative analysis. Six studies presented data related to determinants of suicidality in cluster headache [10–12, 14–16, 18, 19] and were considered for the qualitative analysis.

**Table 2:** Data extracted in selected studies (CH: cluster headache, CCH: chronic cluster headache, C-SSRS: Columbia suicide severity rating scale, ECH: episodic cluster headache, EHR: electronic health records, HIT6: headache impact test, MA: meta-analysis, OR: odds ratio, PHQ9: patient health questionnaire 9 items, SBQ-R: suicidal behavior questionnaire revised)

Ref	Authors	Year	Number Subjects	Suicidality evaluation	Data reported	Suicidal Ideation selected rate for MA	Suicide Attempts selected rate for MA
[10]	Jürgens et al.	2011	75	One question *‘whether you had thought about death and/or experienced suicidal tendencies’* Lifetime evaluation	Response ‘yes’ Total 13/75 17,3% ECH outside-bout 3/22 13.6% ECH in-bout 4/26 15.4% CCH 6/27 22.2%	17.3%	no data available
[11] [12] [14]	Rozen and Fischman	2012 2018	1134	Two questions *‘think about suicide’* *‘try to commit suicide’* Lifetime evaluation	Responses ‘yes’ *‘think about suicide’* n? 55% tobacco exposed vs. tobacco non-exposed 57% vs. 43%, *p* = 0.003 men vs. women 57% vs. 52%, NS *‘try to commit suicide’* n? 2% men vs. women 2% vs. 2%, NS	55%	2%
[13]	Choong et al.	2017	7589	Suicidal-related claims Hospitalization with specific ICD9 codes Suicidal ideation (V6284) Self-inflicted injury (E950-E959) 1 year before 1 year after the first CH claim	Claims Suicidal ideation (V6284) 89/7589 1.2% Self-inflicted injury (E950-E959) 15/7589 0.2%	1.2%	0.2%
[15]	Ji Lee et al.	2019	175	Four questions *‘Have you thought that it was better to die…?’* *‘Have you thought of killing yourself…?’* *‘Have you planned suicide…?’* *‘Have you attempted suicide … ?’* 3 clinical situations: ictal interictal between-bout (only 54 patients) Lifetime evaluation	Responses ‘yes’ *‘Have you thought that it was better to die…?’* passive suicidal ideation ictal: 111/175 64.2% interictal: 7/175 4% between-bout: n? 0% *‘Have you thought of killing yourself…?’* active suicidal ideation ictal: 62/175 35.8% interictal: 6/175 3.5% between-bout: n? 1.9% *‘Have you planned suicide…?’* suicidal plan ictal: 10/175 5.8% interictal:5/175 2.9% between-bout: n? 1.9% *‘Have you attempted suicide …?’* suicidal attempt ictal: 4/175 2.3% interictal: 2/175 1.2% between-bout: n? 0% ictal suicidality associated with CH duration (10-y) OR 1.91 [1.29–2.83] HIT6 score (10-point) OR 3.42 [2.01–5.04] PHQ9 score (10-point) OR 3.37 [1.93–5.91] interictal suicidality associated with lifetime bouts (per 5) OR 1.20 [1.05–1.38] PHQ9 score (10-point) OR 4.62 [1.73–12.33]	64.2%	2.3%
[16]	Koo et al.	2021	100	Suicidality evaluation tools SBQ-R/C-SSRS 4 items evaluated passive suicidal ideation active suicidal ideation suicidal plan suicide attempt Lifetime evaluation	passive suicidal ideation 59/100 59% active suicidal ideation 47/100 47% suicidal plan 25/100 25% ECH vs. CCH 16.1% vs. 36.4%, *p* = 0.02 s uicide attempt 8/100 8% ECH vs. CCH 3.6% vs. 3.6%, *p* = 0.07 suicidal ideation associated with demoralization (OR 6.66 [1.56,28.49]) but not depression (OR 1.89 [0.66,5.46])	59%	8%
[17]	Lainez et al.	2022	233	Suicidality evaluation tool C-SSRS 2 items evaluated suicidal ideation suicidal behavior Lifetime evaluation	suicidal ideation 52/233 22.3% suicidal behavior 9/233 3.9%	22.3%	3.9%
[18]	Kim et al.	2022	445	Two questions ‘*Have you ever thought that* *it was better to die?*’ ‘*Have you ever attempted suicide?*’ Lifetime evaluation	Responses ‘yes’ ‘*Have you ever thought that* *it was better to die?*’ 93/445 20.9% ‘*Have you ever attempted suicide?*’ 4/445 0.9% suicidal ideation significantly associated with CH diagnostic delay (≥ 7 years) suicide attempts not associated with CH diagnostic delay	20.9%	0.9%
[19]	Seng et al.	2024	24,131	Veterans Health Administration EHR data 2 items evaluated suicidal ideation suicide attempt 5 years after CH diagnosis	Positive data suicidal ideation 1556/24,131 6.5% suicide attempt 335/24,131 1.4% suicidal ideation men vs. women 8.9% vs. 6.2%, *p* < 0.001 Suicide attempt men vs. women 2.1% vs. 1.3%, *p* < 0.001	6.5%	1.4%
[20]	Calandre et al.	2024	91	9th item of PHQ9 *‘Thoughts that you* *would be better off dead or hurting yourself in some way?’* lifetime evaluation	Response ‘yes’ *‘Thoughts that you* *would be better off dead or hurting yourself in some way?’* 62/91 68.1%	68.1%	no data available
[21]	Göbel et al.	2025	207	Data of center Kiel headache questionnaire 3 items evaluated occasional suicidal ideation regular suicidal ideation intention to commit suicide lifetime evaluation	Responses ‘yes’ occasional suicidal ideation n? 40.5% regular suicidal ideation n? 7,6% intention to commit suicide n? 1.8%	40.5%	no data available

### Risk of bias of individual studies

Assessment of selected case series [10, 15, 18] using Joanna JBI Appraisal Checklist tool is summarized in Table 3 and assessment of selected surveys [11–14, 16, 19–21] using OCEBM critical appraisal tool is summarized in Table 4. The clinical trial [17] was evaluated individually. Selected studies were not excluded based on their quality appraisal.

**Table 3:** The Joanna Briggs Institute (JBI) critical appraisal tool for case series

Ref	Authors	Were there clear criteria for inclusion?	Was the condition measured in a standard reliable way for all participants?	Were valid methods used for identification of the condition for all participants?	Did the case series have consecutive inclusion of participants?	Did the case series have complete inclusion of participants?	Was there clear reporting in the demographic of the participants?	Were the outcomes of follow-up results of case clearly reported?	Was there clear reporting in the presenting site(s)/clinic(s) demographic information?	Was statistical analysis appropriate?
[10]	Jûrgens et al. (2011)	yes	yes	yes	yes	yes	no	yes	yes	yes
[15]	Ji Lee et al. (2019)	yes	yes	yes	no	no	yes	yes	yes	yes
[18]	Kim et al. (2022)	yes	yes	yes	yes	yes	no	yes	yes	yes

**Table 4:** Oxford centre for Evidence-Based medicine (OCEBM) critical appraisal of survey studies

Ref	Authors (year)	Did the study address a clearly focused question issue?	Is the study design appropriate for answering the research question?	Is the method of selection of subjects clearly described?	Could the way the sample was obtained introduce selection bias?	Was the sample of subjects’ representative with regard to the population to which the findings will be referred?	Was the sample size based on pre-study consideration of statistical power?	Was a satisfactory response rate achieved?	Are the measurements likely to be valid and reliable?	Was the statistical significance assessed?	Are the confidence intervals given for the main results?	Could there be confounding factors that haven’t been accounted for?	Can be results be applied to your organization?
[11]	Rozen Fischman (2012)	yes	yes	yes	no	yes	no	yes	yes	no	no	no	yes
[12]	Rozen Fischman (2012)	yes	yes	yes	no	yes	no	yes	yes	yes	yes	no	yes
[13]	Choong et al.	yes	yes	yes	no	yes	no	yes	yes	yes	yes	no	yes
	(2017)												
[14]	Rozen (2018))	yes	yes	yes	no	yes	no	yes	yes	yes	yes	no	yes
[16]	Koo et al. (2021)	yes	yes	yes	yes	yes	no	yes	yes	yes	yes	yes	yes
[19]	Seng et al. (2014)	yes	yes	yes	yes	yes	no	yes	yes	yes	yes	yes	yes
[20]	Calandre er al (2024)	yes	yes	yes	yes	yes	no	yes	yes	yes	yes	no	yes
[21]	Göbel et al. (2024)	yes	yes	yes	yes	yes	no	yes	yes	no	no	no	yes

### Overall rates of suicidal ideation and suicide attempts in CH

**Fig. 2:**
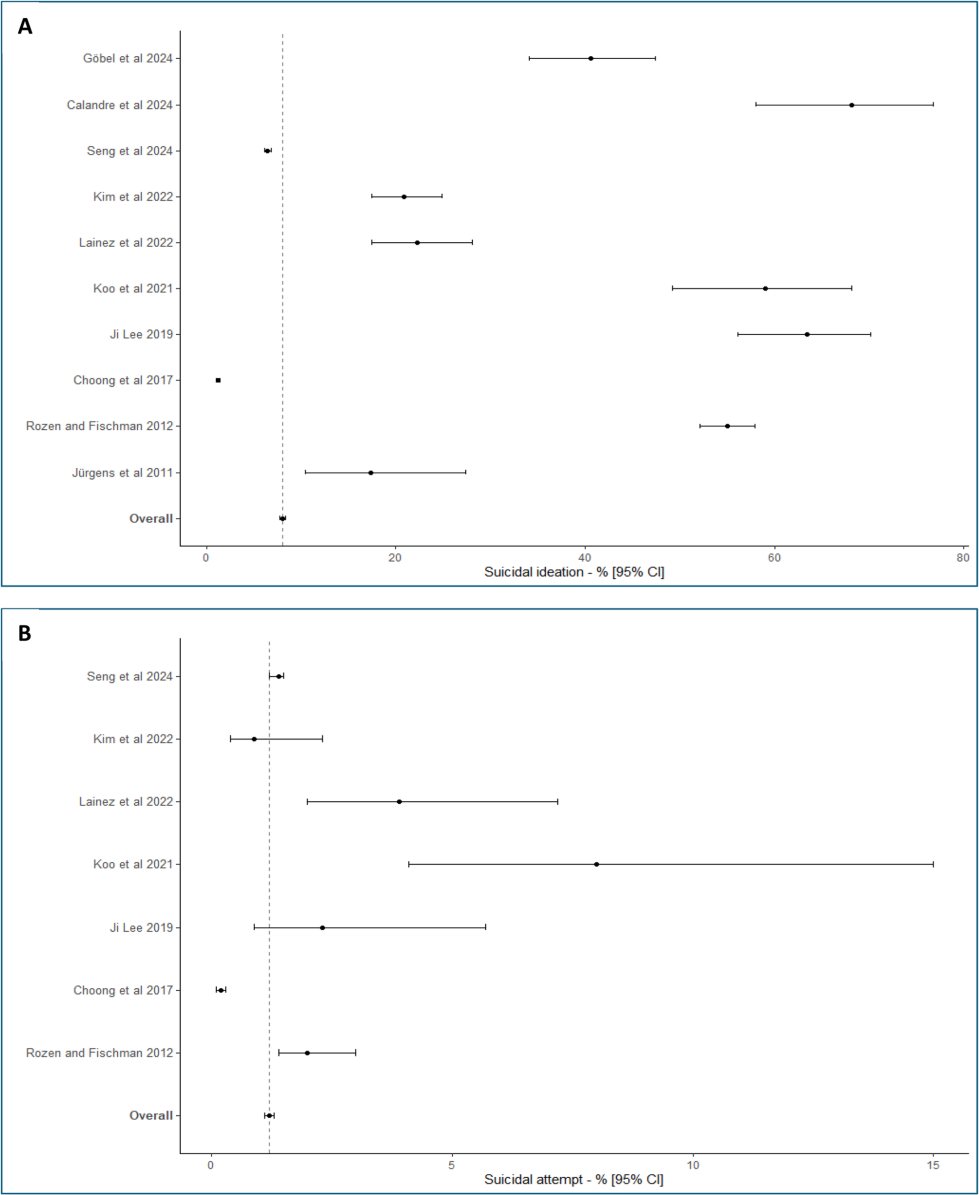
Overall rates of suicidal ideation (**A**) and suicide attempts (**B**) in cluster headache

Considering the ten studies (34180 subjects) for which the rate of suicidal ideation was reported, and which were included in the meta-analysis [10–21], I^2^ was estimated at 99.7%. A Forest-plot of the overall rate of suicidal ideation in CH is presented in Fig. 2. The overall rate of suicidal ideation in CH was estimated at 8.0% (95%CI [7.7; 8.3]).

Considering the seven studies (33807 subjects) included in the meta-analysis [11–19], for which the rate of suicide attempts was reported, the I^2^ was estimated to be 93.8%. Forest-plot of the overall rate of suicide attempts in CH is presented in Fig. 2. The overall rate of suicide attempts in CH was estimated at 1.2% (95%CI [1.1; 1.3]).

### Rates of suicidal ideation and suicide attempts in CH according to studies recruitment

#### Specialized recruitment

**Fig. 3:**
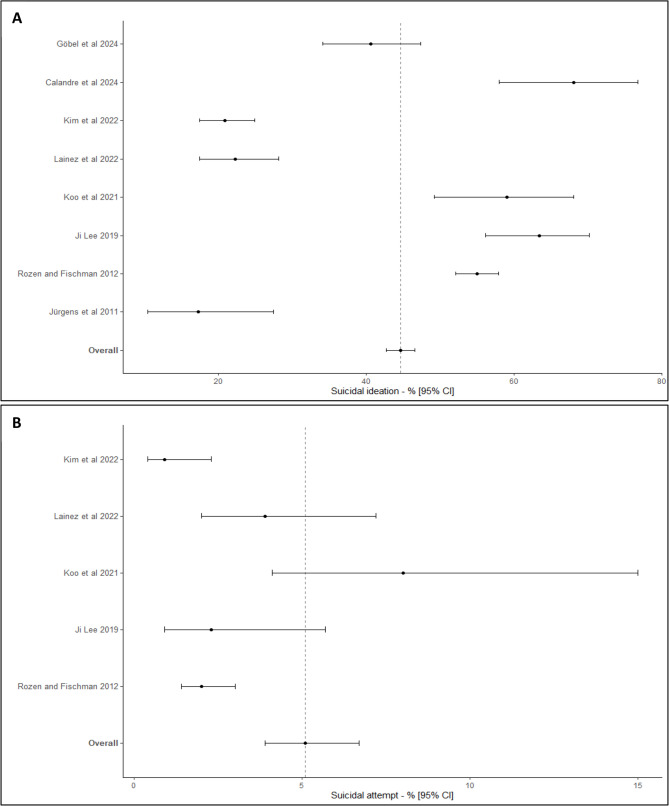
Rates of suicidal ideation (**A**) and suicide attempts (**B**) in cluster headache with a recruitment in specialized field

Considering the 8 studies in which the rate of suicidal ideation was reported, with a specialized recruitment (2460 subjects), and included in the meta-analysis [10–12, 14–18, 20]– [21], I^2^ was estimated to be 97.2%. A Forest-plot of the rate of suicidal ideation in CH reported in specialized fields is presented in Fig. 3A. The rate of suicidal ideation in CH reported in specialized field was estimated at 44.6% (95%CI [42.7; 46.6]).

Considering the 5 studies with specialized recruitment (2087 subjects) for which the rate of suicide attempts was reported and included in the meta-analysis [11]– [12, 14–18], I^2^ was estimated to be 77.5%. Forest-plot of the rate of suicide attempts in CH reported in specialized field is presented in Fig. 3B. The rate of suicide attempts in CH reported in specialized field was estimated at 5.1% (95%CI [3.9; 6.7]).

#### Non-specialized recruitment

**Fig. 4:**
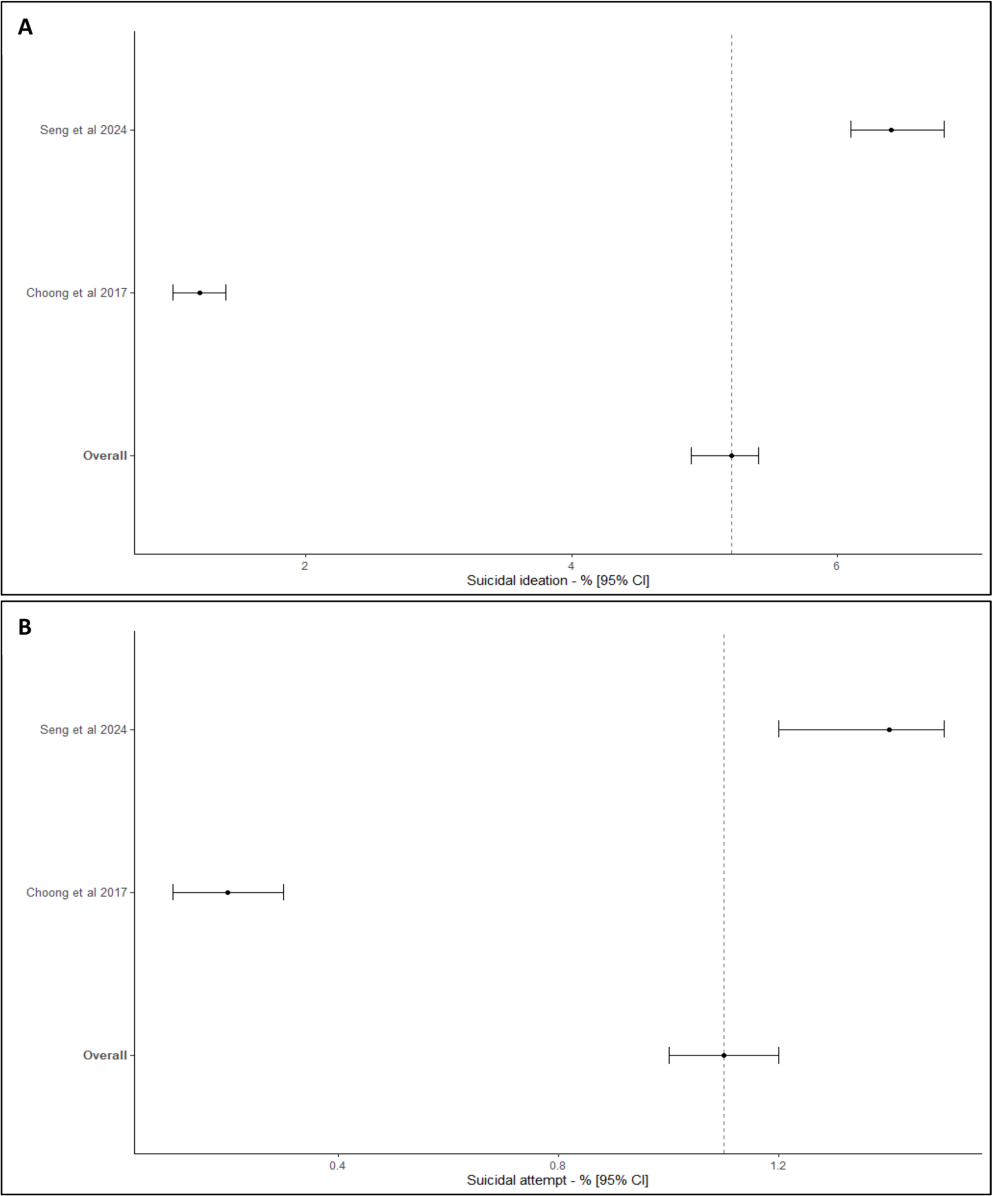
Rates of suicidal ideation (**A**) and suicide attempts (**B**) in cluster headache with a recruitment in non-specialized field

Considering the 2 studies with a non-specialized recruitment (31720 subjects) for which the rate of suicidal ideation was reported, and which were included in the meta-analysis [13, 19], I^2^ was estimated to 99.6%. Forest-plot of the rate of suicidal ideation in CH reported in non-specialized field is presented in Fig. 4A. The rate of suicidal ideation in CH reported in non-specialized field was estimated at 5.2% (95%CI [4.9; 5.4]).

Considering the 2 studies with a non-specialized recruitment (31720 subjects) for which the rate of suicide attempts was reported, and which were included in the meta-analysis [13, 19], I^2^ was estimated to 98.2%. Forest-plot of the rate of suicide attempts in CH reported in non-specialized field is presented in Fig. 4B. The rate of suicide attempts in CH reported in non-specialized field was estimated at 1.1% (95%CI [1.0; 1.2]).

### Comparison of rates of passive suicidal ideation and active suicidal ideation

Ji Lee et al. showed that the rate of active suicidal ideation was lower than the rate of passive suicidal ideation (35.8% vs. 64.8%) in the ictal phase, whereas its rates were similar in the inter-ictal phase (3.8% vs. 4%) [15]. Regardless of ictal and interictal assessment, Koo et al. also found a lower rate of active suicidal ideation than passive suicidal ideation (25% vs. 47%) [16].

### Determinants of suicidality in CH

The influence of gender on suicidality in CH has been evaluated in two studies [12, 19} with contradictory results. Rozen and Fischman found no statistically significant difference in the rates of suicidal ideation and attempted suicide between men and women [12], whereas Seng et al. found significantly higher rates of both in men [19].

The influence of the CH form was evaluated in two studies [10, 16]. Jürgens et al. suggested a higher rate of suicidal ideation in patients suffering from CCH but did not assess its significance [10]. Koo et al. found no difference in the rate of suicide attempts according to the form of CH [16].

Kim et al. found a significant association between the rate of suicidal ideation and the delay in diagnosis (≥ 7 years) but did not find such an association for the rate of suicide attempts [18].

In a detailed study of patients suffering mainly (almost 90%) from ECH, Ji Lee et al. showed that the clinical expressivity of CH influenced the rates of suicidal ideation and suicide attempts, which were higher during attacks than between attacks or during periods of remission [15]. In this work, the authors carried out a study of factors associated with suicidality [15] showing that ictal suicidality was associated with the CH duration, the HIT-6 score and the PHQ-9 score, whereas interictal suicidality was associated with the cumulative number of painful bouts and the PHQ-9 score (detailed data in Table 2).

In another association study, Koo et al. [16] showed that the rate of suicidal ideation was linked to demoralization (according to demoralization diagnostic criteria for use in psychosomatic research and the Kissane Demoralization Scale) and not to depression (detailed data in Table 2).

Finally, Rozen showed an association between the rate of suicidal ideation of CH patients and smoking [14].

## Discussion

Our meta-analysis assessed at the two extremes of suicidal behavior, i.e. suicidal ideation and suicide attempts, observed in CH and was carried out for all studies, for studies performed in a non-specialized field (with a lesser selection bias due to the inclusion of patients of varying severity and being more representative of patients suffering from CH as a whole) and for studies performed in a specialized field (with a selection bias in favor of the most severely affected patients). The overall rate of suicidal ideation in CH was estimated at 8% (95%CI [7.7; 8.3] and the overall rate of suicide attempts in CH was estimated at 1.2% (95%CI [1.1; 1.3]). Meta-analysis performed with studies related to a recruitment in a non-specialized field (which concerned more than 90% of patients included in all selected studies) shows similar results with rate of suicidal ideation and rate of suicide attempts estimated at 5.2% (95%CI [4.9; 5.4]) and 1.1% (95%CI [1.0; 1.2]) respectively. These figures do not seem to indicate a higher overall level of suicidality associated with CH when compared with available epidemiological data on suicidality in the general population. For example, in an analysis of World Mental Health Survey data from 17 countries, the reported cross-national lifetime prevalence (standard error) of suicidal ideation and the reported cross-national lifetime prevalence (standard error) of suicide attempts were 9.2% (0.1) and 2.7% (0.1) respectively [22]. Nevertheless, a recent Danish population-based cohort study, not included in our meta-analysis because it did not individualize patients with CH, showed a suicidal risk increase in subjects diagnosed with TAC (*n* = 6872) compared to matched controls (*n* = 597430) demonstrated by hazards for attempted suicide (HR, 1.97; 95% CI, 1.35–2.87) and for completed suicide (HR, 2.40; 95%CI, 1.23–4.66) [23]. This latest study suggests the need to assess the overall level of suicidality observed in the CH country by country, especially as it has been shown that suicidality in the general population is characterized by great geographical heterogeneity [24].

On the other hand, the meta-analysis carried out on the studies recruited from the specialized field revealed a much higher level of suicidality associated with CH with rate of suicidal ideation and rate of suicide attempts estimated at 44.6% (95%CI [42.7; 46.6]) and 5.1% (95%CI [3.9; 6.7]) respectively. As assumed by the background of our study, such results argue in favor of a suicidal risk which would concern the most severely affected CH patients. Surprisingly, our systematic review did not find any robust data comparing the suicidality of patients suffering from ECH with that of patients suffering from CCH. On the other hand, Ji Lee et al. showed higher rates of suicidal ideation and suicide attempts during CH attacks [15]. Such ictal suicidality may be explained by the extreme severity of the pain, but it could also be part of the behavior observed during CH attacks. Beyond restlessness, which is a diagnostic criterion for CH [1], impulsive self-inflicted injuries have been reported during CH attacks [25]. Such self-aggressiveness could result from the activation of the posterior hypothalamus demonstrated during the CH attack [26]. There is basic evidence for the involvement of the ventro- and dorsomedial as well as the posterior hypothalamus in the pathophysiology of aggressiveness [27] and the posterior hypothalamus has been proposed as a target to treat patients with refractory aggressiveness using hypothalamotomy [28] or deep brain stimulation [29]. Given that the disruption of the hypothalamic–pituitary–adrenal axis plays a key biological role in stress reactivity associated with suicidal behavior [30] and that impulsivity is a component of most psychological models of suicide [31], the impulsive aggressiveness observed in CH attacks could be an important precipitating factor of suicidal behavior observed during the ictal phase of the CH [3].

Caution is also needed when interpreting the relationship between suicidal risk in CH patients and depressive comorbidity, which is three times more frequent in these patients [32]. Overall, the association seems obvious, especially as it has been well demonstrated that depression, like all other psychiatric disorders, increases the risk of suicide [33]. The results of Ji Lee et al. point in this direction, showing a significant association between the PHIQ-9 score and suicidality, whether ictal or interictal [15]. However, in a more detailed study of the psychological determinants of suicidality in CH patients, Koo et al. showed that the rate of suicidal ideation was linked to demoralization (according to demoralization diagnostic criteria for use in psychosomatic research and the Kissane Demoralization Scale) and not to depression [16]. Interestingly, Koo et al. found that suicidal ideation was associated with depression and not to demoralization in controls [16], suggesting that demoralization may be particularly sensitive to suicidality in CH.

Our qualitative analysis has shown that certain determinants of suicidal risk have not been evaluated in studies devoted to this topic. In particular, the association of suicidal risk with sleep disturbances and the use of illicit drugs was not considered, whereas both are pain-related risk factors for suicidality in chronic pain [34]. Sleep disturbances and illicit drug use should therefore be considered in association studies with suicidality, as they are more frequently observed in CH patients than in the general population [35, 36]. It would also be interesting to assess whether cluster headache patients who don’t feel understood by those around them have an influence on their suicidality. In the Eurolight Cluster Headache project, half of cluster headache sufferers reported avoiding telling others about their disease (in particular, subjects with CCH and a high attack frequency as well as those worry about future attacks) and such CH sufferers felt understood by their families and colleagues less frequently [37]. At the very least, feeling understood generates frustration. But it may also be perceived by some patients as bullying, which could increase their behavior of non-suicidal self-injuries and their suicidality [38]. Even if the identification of genes that place people at risk of suicide remains elusive [39], future studies on suicidality in the CH could also associate gene expression levels measurement. Finally, it would be relevant to assess whether the prophylactic treatments used in CH have any impact on the level of suicidality, something that has not yet been studied [40].

Interpretation of this systematic review and the meta-analysis it includes must remain cautious in view of certain limitations. First, this meta-analysis shows a high degree of heterogeneity among selected studies, since I2 is above 75% for all sub-groups of studies considered. It is also important to note that only three studies [16, 17, 20] assessed suicidality using patient reported outcomes tools developed and validated to measure this risk specifically. Finally, the studies carried out in non-specialized fields [13, 19] were based on insurance databases and not on the general population.

## Conclusions

This systematic review suggests an overall suicidal risk in CH that may not be significantly higher than in the general population. Nevertheless, the limitations of the selected studies on suicidality in CH and of our meta-analysis should make us cautious in interpreting these results, pending a comparative study with the general population using dedicated PROs and considering the geographic heterogeneity of suicidality. On the other hand, it showed a clear increase in this risk among CH patients followed up in specialized fields (tertiary care center patients, neurological clinic, patients linked to patients’ associations), with almost half of these patients declaring suicidal ideations and more than 5% having made suicide attempts. This result confirms the need for a systematic assessment of suicidality in patients with cluster headache, particularly those treated at a tertiary headache center. These patients are a priori the most severely affected by CH, with the highest intensity of pain during attacks, the highest number of attacks and significant depressive comorbidity. Nevertheless, the qualitative analysis carried out in this systematic review suggests that the suicidal behavior observed in CH is more complex, probably involving impulsive aggressiveness during attacks and demoralization. Future studies aimed at clarifying the determinants of suicidal risk in CH will need to integrate all these elements, adding sleep disorders and illicit drug use, two potential determinants that have never been considered to date. Such an approach is essential if we are to develop effective suicide prevention for CH patients.

## Data Availability

No datasets were generated or analysed during the current study.
